# COVID-19 and Brazilian Indigenous Populations

**DOI:** 10.4269/ajtmh.20-0563

**Published:** 2020-06-09

**Authors:** Graziela Almeida Cupertino, Marli do Carmo Cupertino, Andréia Patrícia Gomes, Luciene Muniz Braga, Rodrigo Siqueira-Batista

**Affiliations:** 1 School of Medicine, Faculdade Dinâmica do Vale do Piranga, Ponte Nova, Minas Gerais, Brazil;; 2 Department of Medicine and Nursing, Universidade Federal de Viçosa, Viçosa, Minas Gerais, Brazil

## Abstract

The newly discovered SARS-CoV-2 is the cause of COVID-19, including severe respiratory symptoms with an important lethality rate and high dissemination capacity. Considering the indigenous people of Brazil, it is feared that COVID-19 will spread to these communities, causing another stage of decimation. Despite advances in indigenous health care in the country, there are still many challenges due to the social vulnerability of this population, whose lands continue to be illegally exploited. Based on these considerations, this article discusses challenges in caring for the indigenous population in the context of the COVID-19 pandemic in Brazil.

## INTRODUCTION

The indigenous population that inhabited Brazil in 1500 was, according to estimates, around 3 million people strong, distributed throughout the national territory in more than a thousand different ethnic groups. Of these, 2 million occupied the Brazilian coast and the rest the interior. Seventy years after the discovery of Brazilian lands and contact with the indigenous peoples by Europeans, this population was drastically reduced, estimated at 1,200,000 Indians distributed in the country, 200,000 of them on the coast and one million in the interior.^1,2^

For centuries, the indigenous ethnic groups continued to suffer from the decimation of their peoples and, in 1957, reached their lowest number of inhabitants in the country, about 70,000 (5,000 along the coast and 65,000 in the interior). Diseases, wars, persecutions, and socioeconomic ruptures were mainly responsible for this reduction. Various epidemics of infectious and parasitic diseases were most likely the greatest cause of the demographic decline of these peoples.^1,2^

Among the many illnesses that have affected the indigenous Brazilians, from the arrival of the Portuguese to the present day, are helminthiasis, syphilis, gonorrhea, rickettsiosis, tuberculosis, pneumonia, whooping cough, malaria, yellow fever, smallpox, measles, chicken pox, and respiratory viruses.^1^ Pandemic influenza, the Spanish flu, raged during 1918–1919 and was responsible for the deaths of 20 million people around the world. In Brazil, this disease was responsible for the extinction of countless indigenous ethnic groups. All these epidemics and their devastating consequences to the Indian population show the great vulnerability of these peoples to the introduction of new pathogens.^1^

The year 2020 brings a new disease, COVID-19, with high dissemination potential, which has mobilized health authorities worldwide. Since its emergence in China in December 2019, it has rapidly spread throughout the world, and, by May 11, 2020, it had affected 215 countries, with more than 4 million confirmed cases and more than 350,000 deaths as of June 1, 2020.^3^ As in other countries, once it was introduced in Brazil, COVID-19 claimed many victims and raised many questions for its control, especially when dealing with the most susceptible groups, such as Indians. Based on these considerations, we discuss challenges in caring for the indigenous population in the context of the COVID-19 pandemic in Brazil.

## SARS-CoV-2 AND COVID-19: A NEW PANDEMIC IN BRAZIL

Coronaviruses (CoVs) are the second leading cause of the common cold, behind rhinoviruses.^3,4^ The CoVs mostly cause mild respiratory infections. However, the emergence of three new species of CoVs that infect humans has altered this reality, with serious outbreaks of SARS-CoV, Middle East respiratory syndrome, and now COVID-19, caused by SARS-CoV-2.

The newly discovered SARS-CoV-2 is capable of causing severe respiratory symptoms, with an important lethality rate, although most infections are mild to moderate. Infection with this pathogen first appeared in December 2019 in Wuhan, Hubei Province, China. On January 30, 2020, the outbreak was declared a public health emergency of international importance by the WHO, and, on March 11, COVID-19 was characterized by the WHO as a pandemic.^3,5^

In Brazil, the first case of COVID-19 was confirmed on February 26, 2020, but retrospective analyses have suggested that the first cases occurred in January. Considering the growing number of suspect cases and the country’s continental dimension, population density, disparities between rural and urban areas, and great socioeconomic inequality, the current scenario points to a multitude of vulnerabilities for this pandemic. Therefore, there is a need for broad and effective measures to contain the advance of COVID-19 throughout the country.^6,7^

## HEALTH OF INDIGENOUS PEOPLE IN BRAZIL

The Special Secretariat of Indigenous Health (SESAI) of the Ministry of Health, created in 2010, reports the existence of 416 distinct indigenous ethnic groups currently in Brazil.^6^ According to the National Indian Foundation (FUNAI), the latest census conducted by the Brazilian Institute of Geography and Statistics shows an indigenous population of 817,963 distributed throughout the country.^2^ Although Indians are present in all states of the federation, the Northern Region has the largest number, with the largest concentration in the state of Amazonas.^2^

In Brazil, the assistance given to this population is the responsibility of FUNAI. Created in 1967, FUNAI is the official body of the Brazilian state related to the indigenous people, and its mission is to protect and promote the rights of indigenous peoples in the country. In the area of health, SESAI is responsible for coordinating and executing the National Policy for the Health Care of Indigenous Peoples (PNASPI), with the mission of ensuring that indigenous health care is integral, resolute, and humanized. Access to health services for members of the indigenous population, as well as for any Brazilian citizen, is guaranteed, with one of the largest and most complex public health systems in the world.^6^ Healthcare coverage includes 34 Special Indigenous Health Districts, strategically divided by territorial criteria, based on the geographical occupation of indigenous communities: 1,199 basic indigenous health units and 67 indigenous health support houses.^2^

Although advances in indigenous health care in the country are renowned, there are still numerous challenges in working with this population. Whether at the organizational, cultural, or geographical level, there is still much to be done. Even if the current PNASPI contemplates differentiated attention to indigenous populations based on sociocultural diversity and the epidemiological and logistical particularities of these peoples, with the guarantee of completeness of assistance, one can observe that care is still focused on palliative and emergency practices.^9–11^

In addition, despite the growing financial resources made available to implement care, the actions have presented few results in health indicators, reflecting the historical inequalities that mark these peoples in relation to the other inhabitants of Brazil, especially when dealing with the scarcity of demographic and epidemiological data. Another important point concerns social participation, which reflects users’ dissatisfaction with the discontinuity of care, the lack of inputs, equipment limitations, the shortage and high turnover of professionals, and deficits in intercultural dialogues that promote consideration of the traditional knowledge of these peoples. There is still a difficulty of access to health services, both at the level of primary care in the villages and of specialized actions and services requiring medium and high complexity. Thus, these ethnic minorities remain at high risk in the fight against all diseases.^9–11^

## SARS-CoV-2 AND COVID-19: A DANGER TO BRAZILIAN INDIGENOUS PEOPLE

The COVID-19 pandemic brings great concern to the indigenous population of Brazil. This is nothing new, as infectious and parasitic diseases are among those most responsible for deaths among Indians, especially when compared with the rest of the national population.^1^ Outbreaks of respiratory diseases such as H1N1 influenza, in 2009, reinforce the fragility of this portion of the population and suggest that Indians are more susceptible to diseases of the respiratory tract.^1^ Therefore, it should be observed that typical cultural behaviors, such as the sharing of gourds and other household utensils, as well as community housing and diverse hygiene practices justify the fear that COVID-19 may spread widely in these communities, causing another stage of decimation.

In addition to the cultural and behavioral aspects of the indigenous people capable of promoting the dissemination of SARS-CoV-2, there is great fear for the socioeconomic relations of communities with non-natives. Commercial activities in the cities to supply the tribes, without the proper recommended prevention measures, and the proximity of the villages to cities where the virus already circulates, can expedite the dissemination of COVID-19 in indigenous lands and tribes. Furthermore, even with social isolation in different regions of the country, missionary actions, the constant invasions of indigenous lands, nonstop hunting, and exploitation activities by logging companies and mines—especially illegal ones—have not ceased in the country. These and other obstacles that have made it difficult to contain the COVID-19 pandemic increase the burden on governments—and especially on FUNAI—for measures to protect native communities.^6^

On April 11, 2020, SESAI reported the death of two indigenous people by COVID-19 in Manaus: a 44-year-old Kokama indigenous woman hospitalized since February 28 for autoimmune hemolytic anemia and a 78-year-old Ticuna indigenous woman hospitalized for cardiovascular treatment. On May 15, 340 confirmed cases, 159 suspected cases, and 21 deaths from COVID-19 were reported in indigenous peoples. The data show a frightening progression of cases, with great concentration in the northern region, especially in the Upper Solimões River (145 cases and 10 deaths), Manaus (38 cases and one death), and Yanomami territory (19 cases and one death).^8^

In this context, government actions have been directed to expand the capacity to attend to COVID-19 cases and avoid the collapse of the health network, which is imminent. Especially in the capital city of Manaus, the number of people in need of hospitalization is increasing daily, and the health system is already operating at the limits of its capacity. As damage containment measures, by May 11, three multidisciplinary teams of the Brazilian National Health System had already been sent to the capital to reinforce care. There was also availability of an online platform that allows physicians to discuss management of each patient in the intensive care unit with other health professionals. Also ongoing are the expansion of hospital beds; hiring of private beds; increases in the transfer of financial resources; sending of supplies, respirators, molecular tests, and individual protection equipment; construction of a campaign hospital with a capacity of 200 beds; convening of health professionals enrolled in the strategic action *O Brasil Conta Comigo* (“Brazil depends on me”); and intensification of recommendations to the population for the adoption of preventive measures.^8^

Despite these actions, rapid progression to chaos is feared among indigenous people, and this possibility becomes even more worrying, given the high prevalence of risk factors for COVID-19 in this population, such as obesity, hypertension, and diabetes mellitus.^12–14^ This may lead to worse prognosis and increased need for hospitalization and advanced life support, as observed in China and Italy.^15,16^

Added to these factors are the social vulnerability of indigenous peoples in the face of disrespect for the social distancing of thousands of miners, lumbermen, and land invaders who continue to exploit indigenous territory illegally; the lack of a protective policy for indigenous peoples by the federal government^17^; the lack of structure and limited access to health care, especially high complexity care; a communal lifestyle; lack of an adequate drinking water supply system, poor hygiene conditions, and difficult access to soap and alcohol gel; and exposure in urban centers during searches for emergency financial aid offered by the federal government.^18,19^

The disaster chronicle can be properly understood when considering the consequences of the H1N1 pandemic in 2009 in Brazil. On that occasion, 34,506 cases of influenza and 1,567 deaths were recorded among indigenous peoples.^20^ Among the most affected groups were children younger than 5 years, patients with respiratory and metabolic comorbidities, pregnant women, and indigenous people. In Brazil, the epidemiological behavior of H1N1 assumed a seasonal pattern, with the highest number of cases in the South and Southeast regions, where the climate is more temperate.^21^ Although these are the two Brazilian regions with the smallest indigenous population, during the course of H1N1, Indians in these regions were subjected to notoriously higher risks for severe infection, with higher rates of hospitalization and lethality, than the nonindigenous population.^20–22^ Therefore, similar to what occurred with the H1N1 pandemic in 2009, the current prospects for COVID-19 in the indigenous population are of more serious consequence than other segments of the Brazilian population. Statistically, Indians were 4.5 times more affected than others by severe acute respiratory disease during the 2009 pandemic.^23^

Thus, if the strategies to contain the advance of COVID-19 for indigenous peoples are not successful, then it is possible that we will experience another historical tragedy,^24^ especially when considering the propagation speed of the virus, and limitations of the Brazilian health system, with a significant overload of patients and scarce resources.

## CONCLUSION

Indigenous peoples are highly vulnerable to the advancement of SARS-CoV-2 infection. If there is not an immediate implementation of a serious care policy for the Brazilian indigenous people, there will be a real possibility of a new wave of decimation of this population, as thousands of deaths caused by other epidemics throughout history have shown. It is imperative that every effort be made to contain the advance of COVID-19 among Brazilian Indians, otherwise the genocide prophetically announced by the brilliant artist Sebastião Salgado^25^ will come to pass.
